# Interventions Supporting Disclosure Among Children and Adolescents Living with HIV: A Scoping Review and Realist Synthesis

**DOI:** 10.1007/s10461-026-05060-2

**Published:** 2026-02-10

**Authors:** Christina Laurenzi, Cassandra Carels, Damilola Walker, Nicola Willis, Magdalena Barr-DiChiara, Wole Ameyan

**Affiliations:** 1 Institute for Life Course Health Research, Department of Global Health, Faculty of Medicine and Health Sciences, Stellenbosch University, 6023 Clinical Building, Francie van Zijl Drive, Tygerberg, Cape Town, South Africa; 2 UNICEF, New York, USA; 3 Zvandiri, Harare, Zimbabwe; 4 Global HIV, Hepatitis and Sexually Transmitted Infections Programmes, World Health Organization, Geneva, Switzerland

**Keywords:** Disclosure, Disclosure interventions, Child and adolescent HIV, Social support, Rights-based approaches

## Abstract

HIV status disclosure remains a persistent challenge for children and adolescents aged 6–19 years. Disclosure is critical to improving treatment adherence, viral suppression, and psychosocial wellbeing, and promoting informed decision-making. This scoping review aimed to identify evidence on interventions focused on disclosing children’s and adolescents’ HIV statuses to them, and those supporting them with onward disclosure. We conducted a global scoping review to identify and analyze interventions supporting HIV status disclosure to, and by, children and adolescents living with HIV, identifying 25 interventions. Interventions were categorized into (1) disclosure-specific (directly focused on disclosure) and (2) disclosure-inclusive interventions (involving disclosure within broader support). Realist methods were used to evaluate disclosure-specific interventions; narrative synthesis was used for disclosure-inclusive interventions due to their heterogeneity. Disclosure-specific interventions shared common building blocks, including recognition of autonomy and dignity, tools to enhance engagement, and integration with broader health and social support systems. These contributed to improved confidence, communication, and decision-making among children and adolescents. Disclosure-inclusive interventions frequently employed intergenerational and peer support models, alongside health system strengthening strategies. Findings revealed differences in available evidence for disclosure interventions, with limited adolescent-tailored disclosure-specific interventions and few rights-based paradigms. While interventions to promote disclosure and retention in care aimed to work within participants’ support systems, fewer made efforts to bolster these systems or enhance linkages; these approaches require further evidence. Priority actions emerging from the review include enhancing training and education, adopting youth-driven and rights-based approaches, strengthening metrics, and adapting to the evolving HIV landscape.

**Supplementary Information:**

The online version contains supplementary material available at 10.1007/s10461-026-05060-2.

## Introduction

 Recent global funding cuts are placing decades of significant progress against HIV in jeopardy [1, 2], and children and adolescents are disproportionately likely to experience negative outcomes of disappearing funding and dramatic disruptions in care [3, 4]. While these rapid changes threaten systems of prevention and care that until recently may have functioned reasonably well, they are also likely to exacerbate the most persistent challenges in the HIV care cascade. HIV status disclosure is a critical, poorly documented challenge. Disclosure refers to the process of being told one’s HIV status (disclosure *to* individuals previously unaware of their status) as well as communicating one’s own HIV status to others (disclosure *by* individuals about their own status to peers, family members, or partners; referred to as *onwards disclosure*). Being aware of and understanding one’s own status, and being able to share this status with others safely and when ready, is key to closing gaps in prevention and care and enhancing treatment outcomes [5]. Supportive strategies and interventions to improve both types of disclosure have been developed and integrated into HIV care programs globally. However, there is limited recent evidence on the most effective approaches to support HIV status disclosure to, and by, children and adolescents [6].

Disclosing to children and adolescents about their own HIV status is the critical first step to building health literacy and empowering them to participate in health-related decision-making [7]. This capability is essential to lifelong health. Disclosure—a layered, socially complex process—is deeply connected to respect for rights, including dignity, privacy, autonomy, and access to information, including for children and adolescents [8]. It is also clinically significant: key evidence demonstrates the association between delayed disclosure, poor mental health, and virological non-suppression [9].

Effective disclosure support should be attuned to children’s and adolescents’ evolving capacities for decision-making about their own health, their individual circumstances, their social support networks, and potential risks that may follow disclosure [10]. Such support should ideally integrate individual, relational, social, and structural considerations.

When considering disclosing a child’s HIV status to them, parents/caregivers often require tailored support to initiate conversations about HIV with their child [5]. Disclosure can alter caregiver-child relationships, and parents/caregivers may also fear involuntary disclosure of their own HIV status to others [11]. Concerns about how to broach difficult topics in age-appropriate ways are also common, especially as children mature into adolescence [12]. Health providers may lack adequate skills and time to address disclosure with the sensitivity and care required [13]. Reluctance from parents/caregivers, healthcare providers, or both, can prolong the process and further disadvantage children who may be ready to exercise greater autonomy [14].

Onwards disclosure may require a distinct set of conceptual and practical approaches. In considering this process, children and adolescents may lack emotional maturity, skills, or confidence to effectively communicate about HIV [15], and also may anticipate or experience stigma from others. Adolescents may feel newly concerned about the consequences of onwards disclosure as friends and peers become increasingly central in their lives. Unintended, unsafe, or even misjudged disclosure to others may disrupt important relationships, with potential spillover of negative consequences for family members [16]. Stigma enacted in healthcare settings, at school and in other community spaces can be particularly damaging during this time, shaping adolescents’ decisions to disclose. Adolescent girls and gender and sexual minorities in particular may fear for their physical safety following disclosure to intimate partners [17].

These complex considerations can help explain why barriers to HIV status disclosure persist in this age group [13, 18]. Children and adolescents living with HIV may bear additional social stigma-by-association [19]. Disclosure of HIV status may also be accompanied by other disclosures—of parental HIV, childhood trauma or abuse, or declaration of gender identity. Because disclosure is a fluid, iterative process that requires continual adaptation, post-disclosure support is also critical [20]. However, because disclosure happens differently over time with diverse individuals, prior experiences—positive or negative—shape future willingness and intentions to disclose [21, 22]. Decisions about disclosing can be complicated by harmful laws that criminalize non-disclosure or mandate provider notification to authorities [23]. As the implications of funding cuts become clearer among HIV-affected communities, stigma and fear is likely to be amplified, negatively influencing how open people feel to disclose their status [24].

Understanding existing best practices and evidence for disclosure is critical as we move into an uncertain time in the global HIV response. Despite emerging resources that provide guidance on supporting disclosure for children and adolescents specifically [22], there have been no WHO guidelines on how to support adolescents, a priority group, with disclosure. Additionally, the various “directions” of disclosure within programming means that evidence varies across programs including caregiver-to-child disclosure, child-to-child/adolescent-to-adolescent disclosure, and adolescent-to-adult disclosure.

This scoping review aimed to identify key evidence from interventions that focus on disclosing children’s and adolescents’ HIV statuses to them, as well as those supporting them with onwards disclosure. Given the broad nature of this aim, a scoping review was selected to map existing interventions, including grey literature, and identify key gaps for future guidance and interventions. Our review question was “Which existing interventions support disclosure of HIV status to and by children and adolescents living with HIV?”

## Methodology

### Selection Criteria

This scoping review explored existing evidence for interventions supporting disclosure of HIV status to and by children and adolescents living with HIV (see criteria in Table 1). Interventions implemented from 2011 onwards were included, based on the date of the most recent WHO disclosure guidelines for children. Interventions could include programs designed for diverse target audiences, including children and/or adolescents, their parents/caregivers and families, health workers, other adults in their networks, or some combination thereof. Interventions could be delivered on to individuals, pairs, or groups. While we included interventions targeting children and adolescents aged 6–19 years, interventions were included if the mean age of participants fell within this range.

**Table 1:** Selection criteria for review

Area	Specific criteria
Population	∙ Children and adolescents 6–19 years of age living with HIV ∙ Parents/caregivers of children and adolescents ages 6–19 living with HIV ∙ Healthcare providers, including lay health workers, who work with children and adolescents ages 6–19 living with HIV ∙ Other adults working with children and adolescents ages 6–19 living with HIV (e.g. teachers in boarding schools, community organization staff) ∙ Any combination of the above groups
Intervention type	∙ Psychosocial interventions ∙ Educational/literacy interventions ∙ Structural interventions ∙ Structured tools used to guide disclosure
Outcomes of interest	
Primary outcomes	∙ Disclosure of child/adolescent HIV status by parent/caregiver to child/adolescent ∙ Disclosure of child/adolescent HIV status by health worker to child/adolescent ∙ Disclosure by child/adolescent about own HIV status to others, including sexual partners
Secondary outcomes	∙ HIV-related outcomes (testing, self-testing, antiretroviral adherence, viral load suppression, retention in care) ∙ Mental health outcomes including positive mental health (quality of life, self-esteem, functioning), depressive symptoms, anxiety symptoms ∙ Sexual and reproductive health outcomes (condom use, contraceptive use, STIs) ∙ Stigma: internalized, anticipated, enacted stigma ∙ Relationship quality, e.g. caregiver-child communication, partner communication ∙ Health service uptake and satisfaction ∙ Self-efficacy to disclose ∙ Parent/caregiver readiness to disclose their own status ∙ Any potential harms/iatrogenic effects (abuse, intimate partner violence, self-harm) ∙ Consent for HIV testing ∙ Others not listed here

Intervention aims could be specifically focused on achieving an outcome of disclosure, but also on related outcomes (e.g., improved engagement in care, mental health), and foundational skills to promote eventual disclosure (e.g. self-efficacy, communication skills). Intervention types included psychosocial interventions (those using a psychological, behavioral and/or social approach) [25], educational/literacy interventions, and if applicable, structural interventions (e.g. changes in health systems or processes for conducting disclosure). These interventions could be implemented following testing and diagnosis, and/or at distinct points along the HIV continuum. Types of evaluations included systematic reviews and meta-analyses of relevant interventions; interventions evaluated in individual and cluster randomized controlled trials, crossover trials, factorial trials; interventions evaluated in quasi-experimental studies including pre-post studies; and rigorous qualitative studies and process evaluations. Existing disclosure tools with accompanying evaluations were also reviewed, as were publications detailing the development of disclosure interventions. In addition to peer-reviewed literature, we also engaged with unpublished reports, pre-prints, conference proceedings, and other relevant project documentation from key implementers. No geographic or language-specific restrictions were applied. The scoping review protocol was reviewed and approved by members of a WHO technical advisory group with expertise on disclosure.

### Search Strategy

We conducted a series of searches for peer-reviewed publications in June 2024 on four scientific databases: PubMed, Web of Science, Scopus, and PsycInfo. Grey literature (programmatic reports, technical briefs, conference posters/proceedings) was also reviewed. A total of *n* = 2640 records were retrieved; after removing 1194 duplicates, the final sample of records reviewed was *n* = 1446 (See *Supplemental file 1*). Record abstracts were reviewed by two reviewers against a checklist of pre-set criteria.

A list of 20 included interventions was circulated to an expert consultative group, including members of a WHO technical working group, to check for missing interventions; hand-searching and expert consultations identified 3 additional relevant programmatic interventions and 2 published interventions not identified in the search.

### Data Extraction

The lead author extracted data onto a pre-defined template characterizing key information, intervention characteristics, and delivery specifications. Key information included: implementation setting, target age range, target audience, population-specific considerations, disclosure focus area, intended outcomes, intervention core components, facilitator/delivery agent, delivery platform, dosage, frequency, primary/secondary outcomes, and implementation context. A second author reviewed this extraction template for consistency and completeness. While there was no formal quality appraisal conducted, each intervention publication or intervention evaluation (including grey literature) was reviewed for quality of reporting and completeness of information on content, delivery, and measured impact.

### Data Analysis

Data was analyzed using both realist and narrative methods. Realist evaluation methods are increasingly used to assess and refine interventions, including those focused on adolescent HIV outcomes [26]. Realist methods can provide a structure that helps to explain and understand interventions as they are delivered in “real-world” settings by examining the context and the people involved. These approaches also try to elicit mechanisms that explain how different factors can lead to intended outcomes [27].

The lead author generated rich descriptions for each intervention, building on extracted data. In some cases, multiple publications or evidence types were triangulated to better understand how the intervention was ultimately implemented and evaluated. Interventions were grouped into two typologies, based on the characteristics of relevant interventions, “disclosure-specific” and “disclosure-inclusive.” There was no overlap across these two categories.

Realist review methods were applied to a subset of interventions deemed “disclosure-specific” (see Table 2). For each intervention, these rich descriptions informed a series of context-mechanism-outcome (CMO) statements. To build these statements, the lead author began by extracting authors’ explanations or descriptions of candidate theories of how interventions worked (where available), and further observations and assumptions gleaned from evaluations for each “case” (intervention). Identifying theories, inferring pathways, and testing these pathways is a key part of the realist process, captured in our team’s prior work [26].

**Table 2:** Disclosure intervention typologies used

Typology	Description
Disclosure-specific	• Designed specifically for the purpose of promoting and facilitating HIV disclosure to and/or by children and adolescents living with HIV • Often include multiple components, and targeted at parent/caregiver-to-child disclosure support • Examples include facility-linked parent/caregiver support, healthcare worker training, patient centered materials, and health system quality improvement
Disclosure-inclusive	• Broader in nature, and include disclosure as one of several focal areas to support children and adolescents living with HIV and their families • More commonly targeted at adolescents living with HIV but also parents/caregivers, and healthcare workers • Examples include peer support groups, community health worker visiting, parent/caregiver-child workshops, and integrated services for children in school or health facilities

The lead author then combined these CMO statements into a master list, comparing across distinct cases and identifying commonalities and reinforcing themes. The resulting thematic grouping informed a series of summary statements to populate a working theory of how disclosure-specific interventions work in practice, covering contextual factors (C) shaping mechanisms (M) theorized to unlock specific outcomes (O). Summary statements were derived from extracted data from 11 disclosure-specific interventions with sufficient richness and which reported on evaluations. A second author reviewed these emergent, then final, CMO statements, to support generation of the overarching theory for how these interventions work in practice, for whom, and under which circumstances.

The remaining “disclosure-inclusive” interventions (*n* = 14) were broader in nature, making integration into the realist analysis more complex; we use narrative synthesis to describe these interventions.

## Results

In total, 25 interventions were identified. Eligible interventions were grouped into two categories, based on the types of emerging interventions: (1) *disclosure-specific* and (2) *disclosure-inclusive*. These intervention typologies were examined differently based on their diverse profiles. As *disclosure-specific* interventions focused on disclosure as a common outcome/target, a realist analytic lens was applied to analyze these interventions.

### Disclosure-Specific Interventions

The review identified 11 interventions specifically focused on promoting HIV disclosure to and/or by children and adolescents (Table 3). Many of these interventions helped facilitate disclosure of HIV status to a child/adolescent living with HIV. These interventions tended to be staged and developmentally grounded, focusing on assessing readiness, preparing for disclosure, initiating the process, completing disclosure, and providing post-disclosure support; most incorporated children’s support systems and family environments, and were facilitated with the support of health workers. Several interventions reported adherence, viral load, and mental health outcomes, in addition to completed disclosure.

**Table 3:** Overview of included disclosure interventions

Intervention title	Target population; directionality of disclosure	Country of implementation	Overall aim with key information	Type of supporting evidence
Disclosure-specific interventions				
Namibia Ministry of Health and Social Services pediatric HIV disclosure intervention	Health providers, parents/caregivers, children; disclosure to child/adolescent	Namibia	A multi-component intervention to support disclosure to **7–14-year-olds** living with HIV in healthcare facilities, including a 5-chapter child-friendly book with colorful cartoon drawings designed to guide health providers and caregivers in a gradual disclosure process; a tool to assist health providers to assess caregiver and child readiness for full HIV disclosure and systematically work through barriers; a disclosure form to help health providers monitor children’s understanding of their disease status over time; and a health provider training curriculum to support use of these tools. The average time to full disclosure was 2.5 years.	Qualitative, quantitative (trial evidence), process data
Adapted Blasini disclosure model	Health providers, parents/caregivers, children/adolescents; disclosure to child/adolescent	Haiti; Dominican Republic	A multi-component adapted intervention to support disclosure for pairs of youth living with HIV **ages 10–18** and their caregivers, including a multimedia training/support session for healthcare providers who will participate in disclosure; separate pre-disclosure intervention/education sessions describing pediatric cancer, diabetes, and HIV (for youth) and building capacity for disclosure (for caregiver); a scheduled supportive disclosure session; and separate one-on-one post-disclosure support sessions with psychologists for caregivers and youth. Adaptation included a video for youth as well as a colorful picture book as a disclosure support tool. No time scale was provided for average completion of disclosure.	Quantitative (quasi-experimental)
HADITHI disclosure intervention	Disclosure counselors, parents/caregivers, children; disclosure to child/adolescent	Kenya	A culturally adapted, multi-component disclosure intervention for **10–14-year-olds**, including patient-centered materials to guide disclosure, disclosure counselors who conduct intensive counselling (both individual and group-based), and post-disclosure child support groups to supplement usual care resources. Limited information on dosage for counselling and group support was available, however, children and caregivers were followed over 2 years as they were exposed to this package.	Quantitative (trial)
Adapted disclosure education intervention	HIV clinic nurses, parents/caregivers, children/adolescents; disclosure to child/adolescent	Papua New Guinea, Botswana	An intervention to support disclosure of HIV status to children **aged 3–16 years** in a developmentally-appropriate, staged process, following early steps, intermediate steps, advanced steps, and continuous reinforcement. Both children and caregivers are engaged in this process through a combination of group and individualized sessions, and can be part of the ‘partial disclosure’ or ‘full disclosure’ model.	Observational study, intervention description
Sankofa	Adherence and disclosure specialists, parents/caregivers, children/adolescents; disclosure to child/adolescent	Ghana	A disclosure-focused intervention delivered by an adherence and disclosure specialist who is trained to address information, motivation, and behavioral skills of caregivers of children and adolescents living with HIV **ages 7–18**, tailored to their circumstances, to facilitate their engagement in the process of disclosure (i.e., pre-disclosure, disclosure, and post-disclosure phases) in a manner suitable to the age and needs of the child, delivered over time. Progression through the intervention is guided by stage.	Quantitative (trial)
Family group psychotherapy	Parents/caregivers; disclosure to child/adolescent	Italy	A family group psychotherapy intervention, facilitated by psychologists, including 8 × 2 h group sessions once monthly with small groups of caregivers who have a child living with HIV (aged **4–18 years**). The overall aim was to remove barriers that prevent disclosure and work with small groups of caregivers to discuss common problems and feelings in order to build competence and self-reliance in families and patients, so that parents and their children could effectively manage their own health.	Quantitative (trial)
Kalembelembe disclosure program	Health providers, peer providers, children/adolescents; disclosure to child/adolescent	DR Congo	A four-phase hospital-based intervention for adolescents living with HIV **ages 10–19 years**, facilitated by healthcare workers and peers aged 12–24 years. Phases include preliminary preparation, partial disclosure, full disclosure, post-disclosure support groups.	Observational study
Plan-Do-Study-Act cycles	Health providers; disclosure to child/adolescent	United States	A quality improvement intervention targeting healthcare workers in an HIV clinic team working with children **ages 11 and older** to prioritize disclosure and create opportunities for planning and following up on disclosure support. In this iteration, 6 cycles took place and were monitored over 20 months.	Observational study
Quality improvement collaborative	Health providers, parents/caregivers, children/adolescents; disclosure to child/adolescent	Zimbabwe	A quality improvement strategy to actively document and follow-up with adolescents living with HIV (**aged 10–19 years)**, including documentation of disclosure status, monthly caregiver support meetings and individual counselling, health worker initiated opt-out disclosure sessions, and peer support counselling pre and post disclosure. In this iteration, these processes were measured after 6 months.	Quantitative (pre-post)
PHRU disclosure intervention	Parents/caregivers, children/adolescents; disclosure to child/adolescent	South Africa	A disclosure-focused intervention including two standardized disclosure counselling tools (the Right to Care flipster books and USAID/PEPFAR disclosure package) and a disclosure counselling form delivered to caregivers and children **aged 7–13 years**. The intervention took place in 45–60 min sessions over 78 weeks, alternating 6-weekly between disclosure counselling sessions (completed in Week 72) and psychometric sessions (completed in Week 78).	Qualitative (evaluation)
Story: “Peter’s and Julia’s Discovery: conversing about health and illness”	Parents/caregiver, children; disclosure to child/adolescent	Brazil	A storytelling intervention with children supported by a story across a series of meetings with children living with HIV **aged 7–9 years** and caregivers, delivered over 7 months.	Qualitative (evaluation)
Disclosure-inclusive interventions				
Sauti ya Vijana	Adolescents; onwards disclosure	Tanzania	An intervention to support the mental health of adolescents and youth living with HIV **ages 12–24** through 10 modules delivered in person in groups, by near-peer counsellors in health facilities. SYV is based on principles of cognitive behavioral therapy, interpersonal therapy and motivational interviewing.	Quantitative (trial)
KidzAlive	Health providers; disclosure to child/adolescent	South Africa	A multi-component, child-centered capacity building package for healthcare workers supporting children and adolescents living with HIV **ages 0-12 years**. It integrates healthcare worker training, mentorship, a talk tool (storybook) including conversations around HIV and disclosure, and child-friendly spaces to support the wellbeing	Qualitative (evaluation)
Red Carpet Program (RCP)	Health providers, school personnel, peer support, parents/caregivers, adolescents; onwards disclosure and disclosure to adolescent	Kenya	A package of adolescent-specific services and interventions, beginning at the time of HIV diagnosis through the provision of fast-track, peer-supported VIP (very important person) services for adolescents and young adults **ages 15–24 years**.	Qualitative (evaluation, process evaluation)
Standardized Patient clinical training intervention	Health providers; disclosure to child/adolescent	Kenya	A two-day training intervention with didactic and practical components focused on enhancing healthcare workers’ skills to provide adolescent-friendly competencies in HIV care. Professional actors were trained to portray 7 cases of adolescents/young adults in a healthcare encounter (15–25 min each, with 5 min for feedback). Disclosure was one of the health topics covered in these case scripts, and healthcare workers were assessed through video-recorded interactions.	Observational (pre-post)
I ACT	Adolescents; onward disclosure	South Africa	A 6-session intervention driven by healthcare provider to deliver support groups, aimed at reducing loss to follow-up following diagnosis to commencement of ART (pre-ART group) and improving retention in care (ART support group). The intervention is typically delivered by covering one topic per month, including a topic focused on disclosure, for groups of up to 15 **adolescents ages 15–19**.	Qualitative (evaluation)
ZENITH intervention	Parents/caregivers, children/adolescents; disclosure to child/adolescent	Zimbabwe	An intervention to provide community-based support through 12–15 home visits by community health workers (CHWs) to the households of children (**6–15 years)** newly diagnosed with HIV. CHWs are trained to facilitate structured discussions at critical points in a child’s progression through HIV diagnosis, treatment initiation, and long-term maintenance for 72 weeks post HIV diagnosis, and disclosure is one of the focal areas.	Quantitative (trial)
Psychosocial support (PSS) program, Right to Care Mini Flipster	Adolescents; disclosure to adolescents and onwards disclosure	South Africa	A facility-embedded, peer support psychosocial support intervention for adolescents and young adults living with HIV who are not aware of their status between ages 10–24, addressing disclosure, treatment adherence, social support, and HIV treatment literacy. Participants are organized into groups by **age (10–13**,** 14–16**,** and 17–24 years).** The peer supporters use the Flipster facilitation model developed by Right to Care (RTC) to facilitate support group sessions with participants at selected safe spaces.	Qualitative (evaluation)
CHAMP+/VUKA	Parents/caregivers, children/adolescents; onwards disclosure	Thailand, South Africa	A multi-session intervention (11 in Thailand, 10 in South Africa) designed for caregiver-adolescent pairs (**aged 12–16 years**) and centered on a cartoon storybook, to support caregiver-child communication, adherence, and youth development.	Qualitative (intervention development); quantitative (trial)
Timiza Ndoto	Treatment supporters (parents/caregivers, siblings, parents, partners), adolescents; onwards disclosure	Tanzania	An add-on to enhanced adherence counselling for adolescents and youth living with HIV (**aged 10–19 years**) who are not virally suppressed in Tanzania, including a one-day workshop for adolescents and treatment supporters (caregivers, siblings, parents, partners) engaging them in separate and joint sessions to support better adherence.	Qualitative (process evaluation), quantitative (pre-post)
Yika Mpiko	Parents/caregivers, children/adolescents; disclosure to child/adolescent and onwards disclosure	DR Congo	A model that combines case management and workshops to engage unsuppressed children and adolescents living with HIV and their caregivers to improve viral load suppression. The model uses multidisciplinary, facility-based teams (MDT) to address gaps in disclosure, treatment optimization, and viral load suppression in EGPAF-supported healthcare facilities and health zones. Sessions for children and adolescents included both disclosed and undisclosed groups.	Qualitative (evaluation)
Adolescent Transition Package (ATP)	Health providers, adolescents; disclosure to adolescents	Kenya	An intervention combining disclosure and transition tools for healthcare workers to use to support adolescents living with HIV **aged 15–24 years** in the transition to adult care, enhanced by continuous quality improvement cycles in the first 6 months of implementation.	Protocol; quantitative (trial)
Positive STEPS	Adolescents; onwards disclosure	United States	An adolescent-focused multicomponent intervention, adapted from an adult intervention, to support adolescents living with HIV **aged 16–24 years** with treatment adherence through five in-person sessions with a trained counselor. This adaptation incorporated SMS text messages and video vignettes.	Quantitative (pilot trial)
Project ACCEPT	Adolescents; onwards disclosure	United States	A skills-building intervention for newly-diagnosed adolescents and youth (**aged 16–24 years**), covering two individual sessions, 9 gender-specific weekly group sessions, and one final individual session to promote adjustments and improve engagement in medical care.	Quantitative (pilot trial)
SMART Connections	Adolescents; onwards disclosure	Nigeria	A structured support group intervention delivered on social media, via a secret Facebook group, to adolescents living with HIV **ages 15–19 years**, to promote adherence and improve retention in HIV care over 5 sessions.	Quantitative (pilot trial)

### Understanding Disclosure-Specific Interventions: What Works, for Whom, in Which Circumstances?

**Fig. 1:**
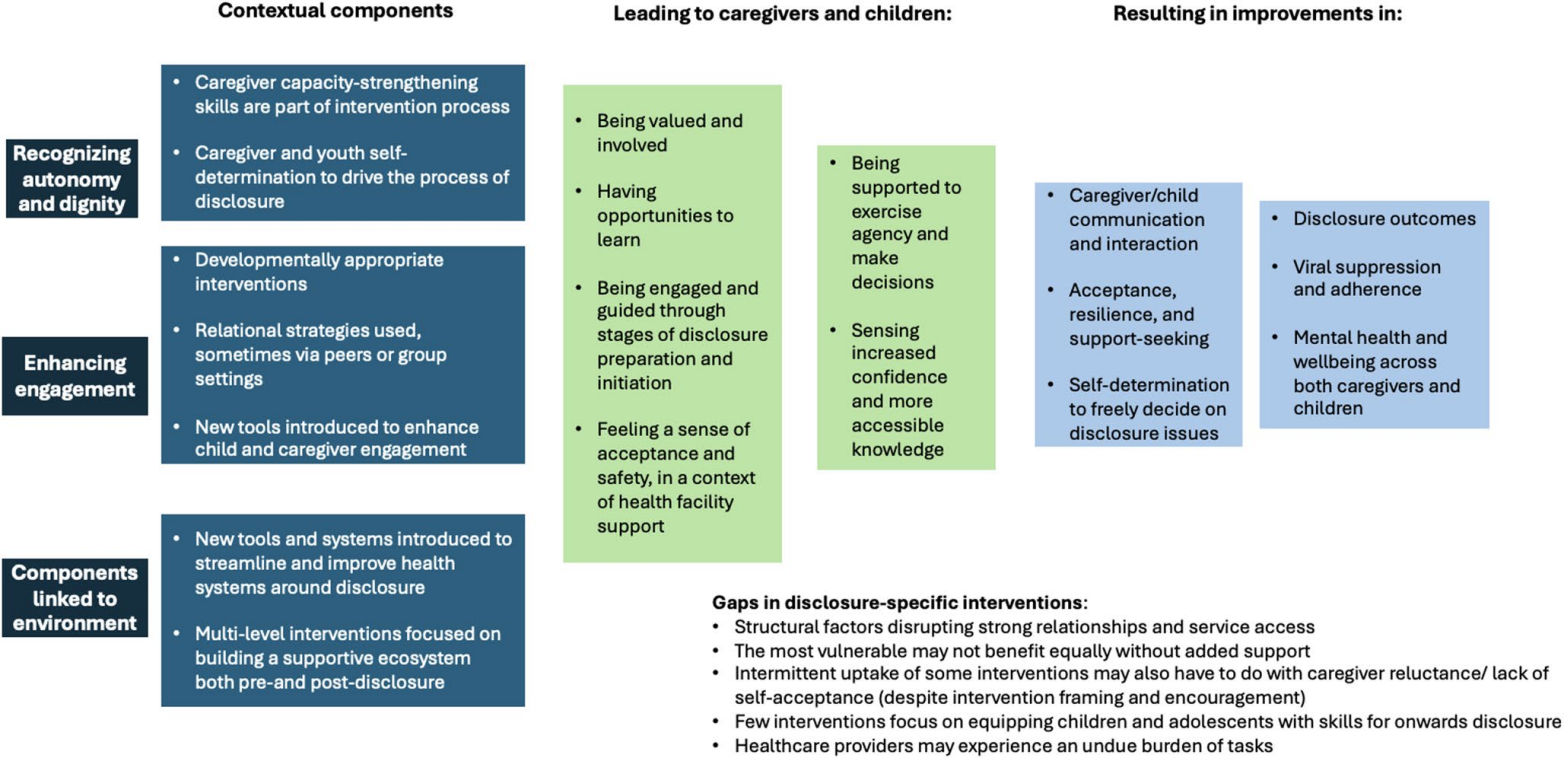
Overarching emerging theory of how disclosure-specific interventions work in practice

Three themes emerged from disclosure-specific interventions: (1) recognizing autonomy and dignity, (2) strategies and tools to enhance engagement, and (3) components linked to broader structures and the environment. Each theme, supported by several CMO statements, supports the overarching theory (Fig. 1).

#### Recognizing Autonomy and Dignity

The first theme links to contextual components of interventions that enable parents/caregivers and children to learn through intervention interactions and feel valued, leading to improved agency, confidence, and preparation for disclosure.

1. When parent/caregiver capacity-strengthening is integrated into intervention content and process (*Context*), parents/caregivers experience opportunities to learn (*Mechanism*), are better able to exercise agency and make decisions (*Mechanism*), and feel more confident (*Mechanism*), leading to improvements in caregiver-child communication and initiation of disclosure (*Outcome*).

For interventions working with parents/caregivers to disclose to children, enhancing confidence and knowledge was critical. While parents/caregivers sometimes became anxious about the process of disclosure and anticipated negative reactions and outcomes [28], they later expressed relief and satisfaction with the guided process. Disclosure interventions enabled parents/caregivers to work with health workers and others to enhance their communication skills and readiness better for difficult conversations [29]. These foundational skills supported improved confidence, setting foundations for disclosure to children.

2. When caregiver and adolescent self-determination are considered important to driving the process of disclosure (*C*), parents/caregivers and adolescents feel valued and become more involved (*M*), are supported to exercise agency and make decisions (*M*), and are able to manage disclosure on their own terms (*O*) with better long-term outcomes on adherence and viral load.

Several interventions provided parents/caregivers and youth with the power and skills to make decisions about the type of disclosure support they wanted [30, 31], and their preference for being involved in the disclosure process [32]. These decisions facilitated more trusting relationships between health workers and parents/caregivers, allowing for knowledge exchange and emotional support, and setting foundations for healthier disclosure. Parents/caregivers were also supported to reflect on their own motivations and the effects of disclosure and non-disclosure in their circumstances [30, 33]. While fewer interventions incorporated elements of adolescent autonomy, in one case, adolescents could invite a trusted friend or family member to join them in a disclosure session [31].

#### Strategies and Tools to Enhance Engagement

The second theme focuses on intervention components that are structured to support children, adolescents, and caregivers to engage in disclosure interventions.

3. Interventions that incorporate developmentally appropriate strategies (*C*) enable parents/caregivers and children to be guided through stages of disclosure readiness and preparation in a way that resonates and feels comfortable (*M*) and support better knowledge of developmental processes (*M*), leading to better mental health and improved timing and suitability of disclosure (*O*).

In many interviews, content was aligned to children’s and adolescents’ developmental stages and readiness for disclosure [29, 30, 34]. Staged approaches to disclosure for parents/caregivers and children often included preparation, partial disclosure, full disclosure, and post-disclosure support; these could be used with adults and children alike to support gradual disclosure [29]. Some intervention content sought to normalize the experience of living with HIV, identify future goals and aspirations, and introduce a sense of hopefulness and positive self-image [29, 31, 35], critical to better disclosure experiences.

4. Relational strategies that enable peer-to-peer engagement as well as group-based support (*C*) lead to a feeling of acceptance and safety (*M*) as well as increased confidence and knowledge (*M*), enhancing the mental health of participants, alongside improved acceptance and confidence with disclosure (*O*).

Peer-based strategies were used both for adults disclosing to children, and for children and adolescents considering onwards disclosure. One holistic, hospital-based model paired trained youth peer supporters with health workers [35]. Peer supporters’ relationships with adolescent patients decreased patients’ sense of isolation; children and adolescents receiving this peer-based approach reported better mental health and adherence compared to those who were disclosed to by health workers or parents. In another group psychotherapy intervention for adult caregivers with a child living with HIV, participants explored barriers to disclosure, learned strategies to enhance competence and self-reliance [36], and practiced skills through role-plays and discussions—ultimately enabling a higher degree of perceived support and increased confidence to initiate disclosure.

5. Interventions that use tools and materials to support child and parent/caregiver participation (*C*) can lead to meaningful, guided engagement (*M*), provide a safe, structured space for enhancing HIV literacy and treatment literacy (*M*), and enhance child and caregiver acceptance as well as facilitate readiness for disclosure (*O*).

Multiple interventions introduced visual aids and multimedia tools to support child and caregiver participation in the disclosure process. These tools included storybooks designed to prepare children for disclosure through understanding HIV disease and treatment [29, 37, 38] as well as other types of visual aids, narrative videos, and animations, or combinations thereof [31, 39, 40]. These tools were used with children and adolescents of diverse ages, as well as their caregivers. One intervention provided health workers with tablet-based resources that were functional offline, catering to children and adolescents in facilities as well as healthcare workers wanting to expand their skillsets [40]. Adolescent participants voiced preference for videos and animations reflecting their own lives, helping increase their self-acceptance following disclosure [39–41].

#### Components Linked to Broader Structures and the Environment

A third and final theme centered on intervention components that worked across multiple socio-ecological levels, creating enabling environments for disclosure. The evidence supporting these structural aspects was largely confined to adult-to-child disclosure pathways.

6. Interventions that introduce tools for streamlining and improving health systems around disclosure (*C*) enhance health workers’ ability to manage patient interactions and frame support for caregivers and children (*M*), leading to better disclosure outcomes (*O*).

Several interventions aimed to refine existing processes and systems to better support health workers with prioritizing disclosure to young patients [32, 42], largely through quality improvement approaches that enabled improvements in documentation and continuity of care, leading to better planning for disclosure. Approaches such as file flags, revised visit note templates for pediatric patients, and routine chart review, tried in multiple iterations, allowed implementers to monitor and revise strategies to enhance their effectiveness in practice. Importantly, these approaches also reduced duplication of efforts and closed gaps in communication, reducing the possibility of unintended disclosure in health facilities.

7. Multi-pronged approaches seeking to create a supportive ecosystem both pre- and post-disclosure (*C*) can increase support for caregivers and children within the health system infrastructure (*M*), and lead to improved resilience, support-seeking, mental health, and disclosure outcomes (*O*).

Several interventions sought to integrate multiple actors and strategies while responding to the patient’s broader environment. Certain approaches prioritized understanding the personal experiences of, and relationship between, caregivers and children in preparing them for disclosure [28]. Health worker support and positive modeling in these cases was critical to facilitating positive disclosure experiences. Using multi-disciplinary teams and/or streamlining strategies resulted in more tailored, client-responsive support [32, 35] and clearer ideas about how to reduce gaps and better prepare at facility level for patient disclosure [42]. Interventions that adopted multiple strategies to tackle disclosure also focused on supporting health worker competencies and knowledge [29, 34], and thereby improving their ability to counsel caregivers and engage with children in a staged disclosure process. Post-disclosure support across several interventions provided additional options for counseling [32, 35] and access to support groups and other social/recreational activities [31].

#### Disclosure-Inclusive Interventions

Fourteen interventions included content and practices supporting disclosure, yet were not solely focused on disclosure (Table 4). In addition to intervention sessions that engaged adolescents in practicing disclosing their own HIV status to others [43–46], evidence described caregiver-child programming that supported intergenerational learning and interaction around the topic of HIV after children and adolescents were aware of their status [47–49]. Several interventions focused on healthcare systems—improving healthcare provider skills [50, 51], health system functioning [52], and health-education integration for adolescents [53, 54] to enable better caregiver-to-child disclosure. These interventions covered a broad set of skills—some linked to disclosure as well as those linked to outcomes that may support disclosure. Many disclosure-inclusive interventions were designed to support longer-term medication adherence by teaching diverse skills and leveraging supportive relationships.

**Table 4:** Description of core components and supporting mechanisms for disclosure-inclusive interventions

Thematic areas	Description of core components across interventions	Mechanisms increasing success of these interventions
Intergenerational approaches [47–49, 73, 74]	∙ Engaging younger and older adolescents living with HIV and their parents and/or caregivers across both joint and separate sessions ∙ Content included parent-child communication, HIV treatment literacy, stigma and discrimination, social support, and preparation for disclosure of the child’s HIV status to others ∙ Use of culturally adapted vignettes/cartoon story	∙ Character relatability; following a story over a multi-session timeline ∙ Supportive group environment, reducing stigma for caregivers and children ∙ Focus on relationships and communication, eventually increasing the willingness and comfort of participating caregiver-child pairs to discuss difficult topics as a family ∙ For shorter interventions, establishing social support networks and decreasing stigma
Peer support approaches [43–45, 75–80]	∙ Often post-disclosure support for older children and adolescents; primarily focused on older adolescents or grouped adolescents by age to ensure age- and developmentally appropriate content and engagements ∙ Diversity of settings and platforms, including governmental health facilities, linkages with community-based organisations, and even digital group-based platforms ∙ Some interventions fully peer-led; others delivered by an adult professional-youth peer team ∙ Often aimed to build skills and confidence of children and adolescents to disclose their status onwards to others	∙ Enhanced peer-peer bonding and acceptance, collective learning and sharing of adherence and disclosure strategies ∙ Reduced stigma and self-stigma ∙ Tailored solutions and situational assessments to prepare adolescents for disclosure to others ∙ Diverse approaches to peer engagement (e.g. through both general open groups in waiting rooms as well as closed groups for adolescents living with HIV) facilitated adolescent choice and supported engagement while also prioritizing their sense of safety and confidentiality.
Health system strengthening [49–52, 74, 75]	∙ Diverse materials developed for use in clinical settings, including a culturally adapted talk tool to prepare children for disclosure ∙ Health provider training covering child and adolescent development, communication skills, empathic skills, technical knowledge of HIV care, supporting children and caregivers, and transition support for adolescents moving to adult care ∙ Home visiting support for recently diagnosed children and adolescents, supporting treatment initiation and retention at household level ∙ Creation of child- and adolescent-friendly spaces in health facilities ∙ Integrating peer supporters into special workshops to bring together children and adolescents alongside caregivers	∙ Improved confidence in communicating with children and adolescents of all ages; improved child-centred approaches ∙ Tailoring content to children, adolescents, and caregivers with diverse relationships ∙ Youth peer educators supporting shifts in framing conversations on disclosure, moving from a focus on medication adherence to future aspirations and ideations ∙ Physical spaces becoming more child-friendly, reducing anxiety and improving health interactions
Broader structural approaches [32, 53, 54]	∙ Included both quality improvement approaches to improve health systems as well as systems for connecting across facility, home, school, and community nodes for children and adolescents ∙ Improvements in documentation, reporting, and key stakeholder communication to identify gaps and enhance processes and flow through system ∙ Multi-stakeholder involvement is also a key innovation, layering HIV and disclosure support across multiple spaces where adolescents are	∙ Reduced gaps in care and partial disclosure ∙ Health provider integration with other systems, staff, and stakeholders ∙ Reducing barriers to retention through bidirectional linkages between schools and health facilities, and clear communications channels ∙ Enabling environments to access support, information, and care

## Discussion

Disclosure as a process carries immense social significance, and the breadth of evidence we reviewed demonstrates its complexity and importance for children and adolescents living with HIV. This review provides updated evidence for both children and adolescents moving across diverse stages of disclosure and onwards disclosure.

Our emerging realist model, drawn from disclosure-specific interventions, focused on internal capacity and autonomy, relational support to enhance engagement, and broader structural and environmental enabling factors. In this discussion, we draw on emerging findings across intervention typologies, and highlight strengths and gaps within this evidence. We close with a recommendations section that outlines areas where interventions could better address the needs of children and adolescents, and key adults in their environments, during pivotal points along their HIV journeys.

### Key Findings and Emergent Gaps

The typology we used to group eligible interventions (disclosure-specific and disclosure-inclusive) revealed key differences in the available evidence for these interventions. As our results revealed, disclosure-specific interventions tended to highlight the role of parents and health workers in disclosing to children, whereas disclosure-inclusive interventions tended to focus on onwards disclosure as well as other key aspects of treatment and care. Practices for disclosing children’s HIV status to them in these interventions were well-informed by evidence [29, 33, 39], and developmental considerations were integrated into several interventions [7]. Many interventions assessed readiness over time with children and adolescents of diverse ages [55], and additional tools provide practical blueprints to facilitate these assessments [5]. Some broader disclosure-inclusive interventions included specific sessions where they discussed support networks, examined anticipated challenges, and shared disclosure experiences [43, 45, 53].

Despite these evidence-based, well-honed approaches, our findings also revealed several important gaps. First, measurement of outcomes was inconsistent. Across child- and adolescent-targeted interventions of all types, better and more consistent measurement of disclosure processes could produce better evidence on disclosure outcomes and processes. While the outcome of full disclosure is critical, supporting factors including child and caregiver HIV literacy, mental health and wellbeing, health worker competency, and caregivers’ disclosure status can be “building blocks” towards this aim [30, 34, 36, 39]. For older children and adolescents weighing onwards disclosure, key factors may include self-efficacy to disclose, self-esteem, and having a support network [56].

Secondly, given the importance of trust-based relationships in supporting healthy disclosure, targeted, ongoing, long-term relationships with care providers is critical. As some mechanisms take longer to take effect, a longer duration of engagement may be important to ensure readiness [29]. Talking with children in ways they can understand—even on a longer timeline—is a priority to ensure timely disclosure. We found limited evidence for these longer-term approaches.

Relatedly, post-disclosure support for children and adolescents is also vital [31], yet onwards disclosure attuned to developmental stage was largely missing from the disclosure-specific interventions we found. After learning about their status, children and adolescents may require further guidance to determine who to disclose to, and how, as new stages of cognitive and social development may bring new understandings about what it means to live with HIV. Transitions to adult care, too, may require that an adolescent fully understands their status [55]. Few documented interventions specifically tackled later-stage disclosure to adolescents, or equipped adolescents to disclose their status onwards. Considering disclosure along the life course means considering how these needs and priorities evolve over time for individuals.

Disclosure is also firmly embedded in conversations about children’s rights [8, 10], yet few interventions expanded beyond youth-sensitive approaches to focus on rights-based or stigma-reduction approaches, or how to navigate previous experiences of unintended disclosure. Interventions should balance children’s rights with the possible constraints that stigma and discrimination may place on children, adolescents, and their families. Negative experiences may delay disclosure or limit onwards disclosure where individuals see few safe alternatives [41, 57], and intersectional stigma may be amplified for individuals with marginalized identities [58, 59].

System-embedded approaches were also limited. While interventions to promote disclosure and retention in care aimed to understand and work within participants’ support systems, fewer actually made efforts to bolster these systems or enhance important linkages. Those that did—such as the Red Carpet Program in Kenya [53]—built long-term connections across schools, health facilities, and households, enabling key individuals in these spaces to support adolescents living with HIV with diverse healthcare needs. These approaches require further attention and evidence.

### Where to From Here? Responding to an Evolving Epidemic and Funding Landscape

In a rapidly shifting global health landscape, disclosure continues to be a cornerstone of HIV prevention and care. However, moving from identifying gaps to offering actionable recommendations is increasingly complicated. The following recommendations attempt to elicit key priorities to help maximize the reach of these actions in an uncertain time for HIV funding.

#### Education and Training

First, education and training remain important priority areas for health providers to facilitate empathic, non-judgmental, stigma-free counselling and disclosure support that includes respect for autonomy. Skills development and technical expertise could enable health workers, HIV care practitioners, and social support staff (social workers, home visitors, and school-based counselors) to differentiate care according to children’s needs and preferences. Training on stigma mitigation should explicitly link with available resources, including other health and social services. Providers should also be aware of legal constraints in their specific contexts. To be optimally responsive to trauma and violence, training should also include knowledge of risk assessment procedures, ensuring that health workers are able to respond to at-risk adolescents with sensitivity, compassion, and professionalism. Support for parents/caregivers, including education on key developmental milestones and capacities that may indicate readiness, is also essential, as is support to develop healthy communication skills and an understanding of additional needs in the post-disclosure period [31, 39].

#### Youth-Directed Decision-Making

Second, encouraging greater youth-directed decision-making could support healthier disclosure and care journeys. While youth have long been visible in HIV programming and advocacy, adolescents need to be involved in conversations around intervention design and delivery [60, 61]. Youth participation may enhance the relevance and acceptability of disclosure interventions. Additionally, embedding peer and lay health providers individuals within multi-disciplinary facility-based teams can increase patients’ access to quality services; mitigate additional burdens for nurses, social workers, and clinicians; increase alignment and skills transfer; and boost motivation. Peer/lay workers should receive initial training and ongoing opportunities for refresher training, mentorship, and supervision to be most effective. Additionally, health workers and implementers should engage directly with adolescents to understand preferences and priorities for new treatment and prevention options and to hear disclosure concerns. This imperative also relies on governments removing punitive policies, including criminalization of HIV status or non-disclosure, so youth feel more able to actively participate.

#### Integrating Routine Indicators into Disclosure

Third, routine indicators collected in healthcare settings could be better framed to capture specific stages of disclosure, providing meaningful, usable data for health providers to learn from. These indicators could be integrated into electronic health records and reporting tools such as DHIS2. Using standardized approaches and definitions can help practitioners and researchers identify patterns across developmental stage and best practices based on specific situations. More rigorous evaluations of cross-sectoral approaches for enhanced adherence and retention in care could bolster evidence about what works for children and adolescents across distinct settings. Improving routine data could also provide critical evidence in the absence of well-funded trials or external research.

#### Engaging Diverse Actors to Support Disclosure

Fourth, engaging actors in children’s and adolescents’ support systems, across the full range of disclosure and service access, can close persistent gaps. Enhanced communication across teachers and administrators in schools, community-based organizations, health providers, and social workers/social welfare is needed, and can encourage better linkages to necessary services. School-based support may include mitigating stigma, but also normalizing testing and counselling for students to reinforce positive messaging [53, 54]. Better, more robust integration across systems can ensure that services are proactive and complementary for children and adolescents along the full continuum of disclosure. Crucially, stringent privacy safeguards and data sharing controls need to be in place. These integrated approaches may also help key adults flag where children may be facing additional struggles, including the structural and social challenges that can make disclosure more complex [31].

#### Responding to an Evolving Epidemic

Finally, advances in HIV prevention, treatment, and care may shift the calculus for how disclosure happens. Advances include service delivery innovations (e.g. multi-month dispensing of ART for virally suppressed patients, including children and adolescents) [62, 63]; novel testing options (e.g. HIV self-testing kits and network-based testing) [64–66]; and treatment options (e.g. long-acting injectable ART) [67, 68]. These options may also limit opportunities for contact with the healthcare system that may support disclosure and post-disclosure processes, or change the urgency for disclosure [69, 70]. Expanded options allow individuals to promote safety with sexual partners while retaining the agency to determine how, and if, to disclose [71]. Adolescents need to be given this information when they are choosing the who, what, and how of disclosure. These advances may benefit individuals living with HIV, as well as complicate well-established modes of disclosure. They also are coinciding with rapid, large-scale disappearance of funding for HIV research and care provision driven by the current US administration. As such, scaling up and intensifying disclosure support programming may rely on integrating these processes into primary care, a process which may require better evidence to effectively implement, as well as better planning of support services and tracking patient needs across clinics and communities.

### Limitations

Despite our best efforts to include all relevant literature, some programmatic examples or publications may have been missed in this review. While realist approaches to analysis may provide more nuance and context specificity than is otherwise possible [72], due to the heterogenous nature of interventions, not all interventions were able to be incorporated into this analysis. Few interventions attended to the multiple disclosures that may be occurring simultaneously, neglecting important opportunities for integration in real-world settings. Complex care arrangements complicate the process of disclosure in many contexts, as children and adolescents move between homes and health facilities, and/or change caregivers. It is not clear that the existing documentation offers a depth of nuance sufficient to address these issues. Lastly, given more time and funding scope, validating the emerging theory with children, adolescents, parents/caregivers, and health workers may have provided greater nuance. These limitations point to critical areas for researchers and policymakers to address in future work on disclosure support for this important age group.

## Conclusion

This paper provides a comprehensive overview of evidence integrated with rights-based approaches that prioritize child and adolescent wellbeing in the process of disclosure, contributing to a critical gap in evidence. Disclosure is both a personal decision and a means to safeguard health and HIV outcomes, especially for younger populations. Because of the diverse ages and stages across which disclosure takes place—and the complex individual and family dynamics that can accompany disclosure—a flexible approach to understanding best practices is needed. Children’s and adolescents’ rights need to be prioritized, alongside emerging social, relational, and systemic considerations that can promote safe disclosure, as shown through our emerging realist theory.

Our findings also point to several key areas for expanding and enhancing systems of support and care. Open-access resources should be prioritized, along with resources that align with existing systems of health education, training, and continuing professional development. Purposeful youth engagement in the context of the current HIV crisis could embolden future leaders in the fields of advocacy, shaping a new form of activism needed to tackle the complexities of the current moment. Alongside better integration of key measures into routine data collection, a stronger emphasis on streamlined systems for care and clear communication channels is increasingly important. At an uncertain moment for global HIV funding, agility and integration of key intervention approaches will become increasingly more central to ensure that children and adolescents living with HIV can lead healthy lives.

## Supplementary Information

Below is the link to the electronic supplementary material.

Supplementary Material 1

Supplementary Material 2

Supplementary Material 3
