# Supplementary material for: Interventions Supporting Disclosure Among Children and Adolescents Living with HIV: A Scoping Review and Realist Synthesis
Source: AIDS Behav. 2026 Feb 10;30(8):2356–72. doi: 10.1007/s10461-026-05060-2 (PMC13486048; doi:10.1007/s10461-026-05060-2)
Supplement: Supplementary file 1 [file 10461_2026_5060_MOESM1_ESM.docx]

## Supplemental file 2: All included interventions, with index publications

| **Intervention title** | **Country where implemented** | **Related published papers** |
| --- | --- | --- |
| Namibia Ministry of Health and Social Services pediatric HIV disclosure intervention | Namibia | Pediatric HIV Disclosure Intervention Improves Knowledge and Clinical Outcomes in HIV-Infected Children in Namibia (Beima-Sofie 2017); “If I Take My Medicine, I Will Be Strong:” Evaluation of a Pediatric HIV Disclosure Intervention in Namibia (O'Malley 2015); Growing-up just like everyone else: key components of a successful pediatric HIV disclosure intervention in Namibia (Brandt 2015) |
| Adapted Blasini disclosure model | Haiti; Dominican Republic | Disclosure of their HIV status to perinatally infected youth using the adapted Blasini disclosure model in Haiti and the Dominican Republic: preliminary results (Beck-Sague 2015) |
| HADITHI disclosure intervention | Kenya | Tablet-based disclosure counselling for HIV-infected children, adolescents, and their caregivers: a pilot study (McHenry 2018); Evaluating a patient-centred intervention to increase disclosure and promote resilience for children living with HIV in Kenya (Vreeman 2019); Factors associated with caregiver compliance to an HIV disclosure intervention and its effect on HIV and mental health outcomes among children living with HIV: post-hoc instrumental variable-based analysis of a cluster randomized trial in Eldoret, Kenya (Magill 2023) |
| Clinic education model for HIV-infected children - Adapted disclosure education intervention | Papua New Guinea | Human immunodeficiency virus status disclosure and education for children and adolescents in Papua New Guinea (Orelly 2018) |
| Sankofa | Ghana | Clinic-Based Pediatric Disclosure Intervention Trial Improves Pediatric HIV Status Disclosure in Ghana (Paintsil 2020); SANKOFA: a multi-site collaboration on paediatric HIV disclosure in Ghana (Reynolds 2015); Pediatric HIV Disclosure Intervention Improves Immunological Outcome at 48 Weeks: The Sankofa Trial Experience (Shabanova 2023) |
| Family group psychotherapy | Italy | Family group psychotherapy to support the disclosure of HIV status to children and adolescents (Nicastro 2013) |
| Kalembelembe disclosure program | DRC | HIV Disclosure to Infected Children Involving Peers: A New Take on HIV Disclosure in the Democratic Republic of Congo (handsearched; Kitelele 2023) |
| Plan-Do-Study-Act cycles | US | Optimizing Disclosure of HIV Status to a Diverse Population of HIV-Positive Youth at an Urban Pediatric HIV Clinic (Dantuluri 2021) |
| Quality improvement collaborative | Zimbabwe | Implementation of a Quality Improvement Collaborative of HIV Status Disclosure of Young People Living with HIV in Zimbabwe (supplied by partner; Sellberg 2023; poster) |
| PHRU disclosure intervention | South Africa | Guiding caregivers through disclosure: a qualitative investigation into caregivers’ responses to a disclosure intervention for HIV-infected children in Soweto, South Africa (handsearched; Joyce 2021; pre-print); Experiences of South African caregivers disclosing to their children living with HIV: Qualitative investigations (handsearched; Joyce 2022) |
| Story: "Peter's and Julia's Discovery: conversing about health and illness" | Brazil | A story for children to help children with HIV understand the health-disease process (Brondani 2013) |
| Sauti ya Vijana | Tanzania | Building resilience: a mental health intervention for Tanzanian youth living with HIV (Dow 2019); A group-based mental health intervention for young people living with HIV in Tanzania: results of a pilot individually randomized group treatment trial (handsearched; Dow 2020); Sauti ya Vijana (SYV; The Voice of Youth): Longitudinal Outcomes of an Individually Randomized Group Treatment Pilot Trial for Young People Living with HIV in Tanzania (handsearched; Dow 2022) |
| KidzAlive | South Africa | Post-training and mentorship experiences of KidzAlive-trained health workers at primary healthcare facilities in KwaZulu-Natal, South Africa (Mutambo 2020) |
| Red Carpet Program (RCP) | Kenya | Optimizing linkage to care and initiation and retention on treatment of adolescents with newly diagnosed HIV infection (handsearched; Ruria 2017); Supporting adolescents living with HIV within boarding schools in Kenya (Kose 2021); Experiences of adolescents and youth with HIV testing and linkage to care through the Red Carpet Program (RCP) in Kenya (handsearched; Kose 2024) |
| Standardized Patient clinical training intervention | Kenya | Pilot evaluation of a standardized patient actor training intervention to improve HIV care for adolescents and young adults in Kenya (Mugo 2019) |
| I ACT | South Africa | Integrated access to care and treatment (I ACT) support groups for adolescents living with HIV in public healthcare facilities in South Africa: feasibility and acceptability for scaling up (James 2018) |
| ZENITH intervention | Zimbabwe | Community health worker support to improve HIV treatment outcomes for older children and adolescents in Zimbabwe: a process evaluation of the ZENITH trial (Dziva Chikwari 2018) |
| Psychosocial support (PSS) programme, Right to Care Mini Flipster | South Africa | Psychosocial Support Programme Improves Adherence and Health Systems Experiences for Adolescents on Antiretroviral Therapy in Mpumalanga Province, South Africa (Okonji 2022); Implementation of a Psychosocial Support Intervention for Adolescents on Antiretroviral Treatment: Challenges and Experiences from Ehlanzeni District, South Africa (Okonji 2022); Applying the biopsychosocial model to unpack a psychosocial support intervention designed to improve antiretroviral treatment outcomes for adolescents in South Africa (Okonji 2022) |
| CHAMP+/VUKA | Thailand, South Africa | Cultural Adaptation of an Evidence-Informed Psychosocial Intervention to Address the Needs of PHIV+ Youth in Thailand (Pardo 2017), The VUKA Family Program: Piloting a family-based psychosocial intervention to promote health and mental health among HIV infected early adolescents in South Africa (handsearched; Bhana 2014) |
| Timiza Ndoto | Tanzania | Timiza Ndoto (Achieving Dreams): Supporting Adolescents and Treatment Supporters in Achieving Viral Suppression (supplied by partner; PEPFAR, USAID, EGPAF, Engender Health; brief) |
| Yika Mpiko | DRC | Optimizing Viral Load Re-suppression and Disclosure Support for Children and Adolescents Living with HIV in the Democratic Republic of the Congo: The Yika Mpiko Model (supplied by partner; EGPAF, 2023) |
| Adolescent Transition Package (ATP) | Kenya | Transition to independent care for youth living with HIV: a cluster randomised clinical trial (Njuguna 2022); Adolescent transition to adult care for HIV-infected adolescents in Kenya (ATTACH): Study protocol for a hybrid effectiveness-implementation cluster randomised trial (Njuguna 2020) |
| Positive STEPS | US | Positive Strategies to Enhance Problem-Solving Skills (STEPS): A Pilot Randomized, Controlled Trial of a Multicomponent, Technology-Enhanced, Customizable Antiretroviral Adherence Intervention for HIV Infected Adolescents and Young Adults (Mimiaga 2019) |
| Project ACCEPT | US | Stigma Reduction in Adolescents and Young Adults Newly Diagnosed with HIV: Findings from the Project ACCEPT Intervention (Harper 2014) |
| SMART Connections | Nigeria | An Online Support Group Intervention for Adolescents Living with HIV in Nigeria: A Pre-Post Test Study (Dulli 2018) |
