# Supplementary material for: Interventions Supporting Disclosure Among Children and Adolescents Living with HIV: A Scoping Review and Realist Synthesis
Source: AIDS Behav. 2026 Feb 10;30(8):2356–72. doi: 10.1007/s10461-026-05060-2 (PMC13486048; doi:10.1007/s10461-026-05060-2)
Supplement: Supplementary file 3 [file 10461_2026_5060_MOESM3_ESM.docx]

**Supplemental file 1. PRISMA flow diagram of sources identified and screened**

**Identification of studies via databases**

Records removed *before screening*:

Duplicate records removed (n = 1194)

Records identified from*:

PubMed (n = 1780)

PsycINFO (n = 59)

Scopus (n = 515)
Web of Science (n = 275)

Hand-searching (n = 11)

**Identification**

Records screened

(n = 1446)

Records excluded

(n = 1399)

Reports sought for retrieval

(n = 84)

Reports not retrieved

(n = 0)

**Screening**

Reports excluded:

Wrong intervention type (n = 10)

Wrong population (n = 14)

Wrong study design (n = 9)

Wrong publication type (n = 10)

Wrong outcome (n=1)

Reports assessed for eligibility

(n = 84)

Studies included in review

(n = 40), belonging to n=25 interventions

**Included**
